# Atrophic Gastritis: A Related Factor for Osteoporosis in Elderly Women

**DOI:** 10.1371/journal.pone.0101852

**Published:** 2014-07-08

**Authors:** Hye Won Kim, Yang-Hyun Kim, Kyungdo Han, Ga Eun Nam, Gwang Seon Kim, Byoung-Duck Han, Anna Lee, Ji Yong Ahn, Byung Joon Ko

**Affiliations:** 1 Department of Family Medicine, College of Medicine, Korea University, Seoul, Korea; 2 Department of Biostatistics, College of Medicine, Catholic University, Seoul, Korea; 3 Department of East Asian Languages, College of Language, Linguistic, and Literature, University of Hawaii at Manoa, Honolulu, Hawaii, United States of America; 4 Department of Internal Medicine, Asan Medical Center, University of Ulsan College of Medicine, Seoul, Korea; 5 Division of Endocrinology, Diabetes, and Metabolism, Beth Israel Deaconess Medical Center, Harvard Medical School, Boston, Massachusetts, United States of America; University of Florida, United States of America

## Abstract

**Purpose:**

Osteoporosis poses a great threat to the aging society. Hypochlorhydric or achlorhydric conditions are risk factors for osteoporosis. Atrophic gastritis also decreases gastric acid production; however, the role of atrophic gastritis as a related factor for osteoporosis is unclear. We investigated the relationship between atrophic gastritis and osteoporosis in postmenopausal women over 60 years of age.

**Subjects and Methods:**

A total of 401 postmenopausal women were included in this cross-sectional study, which was conducted during their medical check-ups. Bone mineral densitometry was measured using a dual energy X-ray absorptiometry. Atrophic gastritis was defined endoscopically if gastric mucosa in the antrum and the body were found to be atrophied and thinned and submucosal vessels could be well visualized.

**Results:**

The proportion of people with atrophic gastritis was higher in the osteoporotic group than in the group without osteoporosis. A linear relationship was observed in the proportion of atrophic gastritis according to the categories of normal, osteopenia, and osteoporosis at the lumbar spine (p for trend = 0.039) and femur (p for trend = 0.001). A multiple logistic regression analysis revealed that the presence of atrophic gastritis was associated with an increased odds of osteoporosis after adjusting for age, body mass index, triglyceride, high-density lipoprotein cholesterol, alcohol consumption, and smoking status (odds ratio 1.89, 95% confidence interval 1.15–3.11).

**Conclusions:**

Atrophic gastritis is associated with an increased likelihood of osteoporosis in Korean elderly women.

## Introduction

Osteoporosis is a metabolic bone disease characterized by the decrease in bone mass with microarchitectual disruption and enhanced skeletal fragility, resulting in an increase for fracture risk [1]. Osteoporotic fractures cause disability and a substantial burden to the society due to both loss of labor and increases in medical expenses. An estimated nine million osteoporotic fractures occurred worldwide in 2000; of these, 1.6 million were hip fractures, 1.7 million occurred in the forearm, and 1.4 million were vertebral fractures [2]. Fragility fractures accounted for 0.83% of the global burden associated with noncommunicable diseases. Osteoporotic fractures contributed to more disability-adjusted life years lost than the common cancers, except for lung cancer, in Europe [2].

The incidences of osteoporosis and osteoporotic fractures are greater in women than in men [3], and bone mineral density decreases according to age [4]. The other risk factors for osteoporosis include cigarette smoking [5], excessive alcohol consumption [6], vitamin D deficiency [7], and low dietary calcium [8]. Calcium is ionized in acidic conditions and absorbed in the small bowel. Therefore, in either hypochlorhydric or achlorhydric stomachs, calcium absorption is impaired [9]. Conditions causing a decrease in gastric acid secretion status, including gastric surgery, and use of proton pump inhibitors increase the risk for low bone mass or fractures [10], [11]. Atrophic gastritis, another hypochlorhydric condition, can adversely affect bone mineral density; however, studies about atrophic gastritis and bone mineral density are sparse and inconclusive [12], [13]. Moreover, to the best of our knowledge, no study has evaluated this association in the elderly over 60 years of age.

The aim of this study was to investigate the relationship between atrophic gastritis and osteoporosis in postmenopausal women aged 60 or older.

## Materials and Methods

### Study subjects

Participants in this study had undergone routine health check-ups at the Center for Health Promotion in the Korea University Anam Hospital located in Seoul, Korea between March 1, 2007 and March 31, 2009. A total of 12,593 persons were examined during this period. Men (n = 6801), persons below 60 years of age (n = 4783), pre- or peri-menopausal women or those with unknown menopausal status (n = 238), persons who had taken drugs that can affect bone mineral density such as glucocorticoids, estrogen, calcium, vitamin D, or bisphosphonates (n = 197), persons who were not examined with dual energy X-ray absorptiometry (n = 137), persons who were not examined with esophagogastroduodenoscopy (n = 30), those with history of gastric surgery (n = 2), and those whose endoscopic biopsy result was dysplasia (n = 4) were excluded from this study. The final study sample had a total of 401 postmenopausal women aged 60 or older. All participants signed the consent form and the Institutional Review Board at the Korea University Anam Hospital approved this study (IRB No. AN09141-001).

### Anthropometric and laboratory measurements

All participants wore light clothing without shoes during anthropometric measurement. Height and weight were estimated to the nearest 0.1 cm and 0.1 kg, respectively. Body mass index (BMI) was calculated using the following equation: weight (kg) divided by the square of height (m). Blood pressure was measured on the upper arm after 10 min of rest using an automated blood pressure monitoring device (MP800, MEKICS, Chuncheon, Korea).

Blood samples were obtained after overnight fasting for 10 h between 08∶00 h and 09∶00 h. An automated analyzer (TB200FR; Toshiba Co., Otawara, Japan) was used to analyze serum total cholesterol (TC), triglycerides (TG), low-density lipoprotein cholesterol (LDL-C), high-density lipoprotein cholesterol (HDL-C), fasting blood glucose (FBG), and uric acid concentration. Hemoglobin level was measured using flow cytometry (XE2100; Sysmax, Kobe, Japan).

### Endoscopic examination and histologic assessment

Biennial gastric cancer screening has been recommended for individuals 40 years and older because of the high prevalence of gastric cancer in Korea, with either an upper gastrointestinal series or endoscopy [14]. Standardized esophagogastroduodenoscopy (GIF-H260, Olympus Co., Tokyo, Japan) was performed by one of two experienced endoscopists at Korea University Anam Hospital, each of whom had at least 5 years of endoscopic experience with over 10,000 cases. Endoscopic findings were described by the overall impression regarding the presence of gastritis in the antrum and the body of the stomach. Atrophic gastritis was defined endoscopically if gastric mucosa in the antrum and the body were atrophied and thinned and submucosal vessels could be well visualized. A single highly experienced endoscopist (JYA) reviewed all endoscopic images, and the diagnosis was confirmed after careful evaluation. A single pathologist (CHK), who was unaware of the clinical details, completed the histologic assessment. The presence of *Helicobacter pylori* was assessed by hematoxylin and eosin and cresyl-violet staining based on the Updated Sydney System [15]. Only a subset of participants (n = 130) underwent *H. pylori* testing.

### Bone mineral density

Bone mineral density (BMD) (g/cm^2^) of central skeletal sites (lumbar spine, total hip, and femoral neck) was evaluated using dual energy X-ray absorptiometry (Discovery-W, Hologic, Bedford, MA, USA). Lumbar spine BMD was measured using the average value for L1 to L4. Femur BMD was chosen as the lowest value between total hip and femoral neck BMD. Osteopenia or osteoporosis was diagnosed using World Health Organization criteria (–2.5<T-score<–1.0 or T-score≤–2.5).

### Statistical analysis

SPSS version 12.0 (SPSS Inc., Chicago, IL, USA) was used for statistical analysis. Results are presented as means ± standard deviation, median with interquartile range, or frequencies and percentages. p<0.05 was considered to be statistically significant. Student t-tests, Mann-Whitney U test, chi-square tests, or Fisher’s exact tests were used to compare the anthropometric, laboratory, social, and endoscopic differences according to the presence of osteoporosis. The chi-square test was used to compare the proportion of atrophic gastritis according to three groups of BMD at the lumbar spine and femur and the linear trend was calculated by using the linear-by-linear association. A multiple logistic regression analysis was performed to assess the association between atrophic gastritis and osteoporosis. Variables that had significant association (p<0.05) with the dependent variable (osteoporosis) in univariate analysis or known risk factors for both osteoporosis and atrophic gastritis were included in the model as covariates. Initially, the analysis was performed without adjustment. Then, age and BMI were adjusted in model 2. In model 3, age, BMI, TG, and HDL-C were adjusted. Lastly, in addition to the covariates in model 3, alcohol consumption and smoking status were adjusted in model 4.

## Results

**Figure 1:**
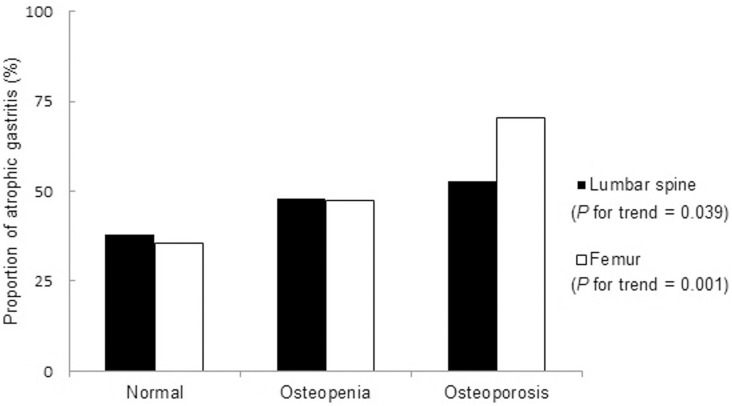
Proportion of atrophic gastritis in three groups of BMD at the lumbar spine and femur. BMD, bone mineral density.

The clinical and biochemical characteristics of the study subjects are presented in Table 1. Osteoporotic patients were older (66.0 versus 63.0 years), had lower BMI values (23.4 versus 24.5 kg/m^2^), higher TG (129.5 versus 121.0 mg/dL), and lower HDL-C levels (50.0 versus 52.0 mg/dL) than subjects without osteoporosis. The proportion of people with atrophic gastritis was higher (56.9 versus 43.1%) in the osteoporotic group than in the group without osteoporosis. The percentage of *H. pylori* infections was greater in the osteoporosis group than in the non-osteoporotic group; however, the difference was not significant. Figure 1 describes the trend of an increasing percentage of atrophic gastritis in the 3 groups of BMD. A linear relationship was observed in the proportion of atrophic gastritis according to the categories of normal, osteopenia, and osteoporosis at the lumbar spine (p for trend = 0.039) and femur (p for trend = 0.001). A multiple logistic regression analysis demonstrated that subjects with atrophic gastritis had an increased likelihood of osteoporosis even after adjusting for age, BMI, TG, HDL-C, alcohol consumption, and smoking (model 4, OR 3.10, 95% CI 1.44–6.68) at the femur (Table 2). A similar result was observed after adjustment was made for anthropometric, laboratory, and social parameters when the dependent variable was regarded as the presence of osteoporosis at the lumbar spine or femur (model 4, OR 1.89, 95% CI 1.15–3.11).

**Table 1:** Clinical and biochemical characteristics of the study subjects.

	Osteoporosis		*P* value^a^
	Yes (n = 102)	No (n = 299)	
Age (yr)	66.0 (62.0–70.0)	63.0 (61.0–67.0)	<0.001^b^
BMI (kg/m^2^)^c^	23.4 (22.2–25.0)	24.5 (22.6–26.2)	<0.001
SBP (mmHg)	126.7±14.7	123.8±13.7	0.075
DBP (mmHg)	70.9±10.3	69.3±10.2	0.169
Hemoglobin (g/dL)	13.3±1.1	13.3±1.1	0.738
TC (mg/dL)	191.3±37.1	191.4±32.5	0.776
TG (mg/dL)^c^	129.5 (93.5–198.3)	121.0 (89.0–168.0)	0.028
HDL-C (mg/dL)^c^	50.0 (42.0–59.0)	52.0 (44.0–61.0)	0.018
LDL-C (mg/dL)^c^	118.0 (100.5–138.3)	115.0 (96.0–138.0)	0.732
FBG (mg/dL)^c^	93.0 (87.8–101.0)	92.0 (86.0–100.0)	0.148
Uric acid (mg/dL)^c^	4.4 (3.9–5.1)	4.5 (3.9–5.2)	0.434
Alcohol drinker	9 (8.9)	30 (10.2)	0.707
Current smoker	3 (3.0)	11 (3.7)	1.000
Atrophic gastritis	58 (56.9)	129 (43.1)	0.016
*H. pylori* infection^d^	26 (61.9)	45 (51.1)	0.249

All values are means ± SD for continuous parametric variables, median (interquartile range) for continuous nonparametric variables, or n (%) for categorical variables. Osteoporosis was defined as a T-score≤–2.5 at the lumbar spine or femur. BMI, body mass index; SBP, systolic blood pressure; DBP, diastolic blood pressure; TC, total cholesterol; TG, triglyceride; HDL-C, high-density lipoprotein cholesterol; LDL-C, low-density lipoprotein cholesterol; FBG, fasting blood glucose.

a Calculated by t-test, chi-square test, or Fisher’s exact test.

b Calculated by Mann-Whitney U test.

c Log-transformed before analysis.

d n = 130.

**Table 2:** Relationship between atrophic gastritis and osteoporosis.

	Lumbar spine		Femur		Lumbar spine or Femur	
	OR (95% CI)	P value	OR (95% CI)	P value	OR (95% CI)	P value
Model 1^a^	1.38 (0.86–2.22)	0.188	2.98 (1.43–6.21)	0.004	1.74 (1.10–2.74)	0.017
Model 2^b^	1.31 (0.80–2.15)	0.278	2.93 (1.38–6.21)	0.005	1.70 (1.06–2.72)	0.027
Model 3^c^	1.33 (0.80–2.21)	0.277	2.94 (1.38–6.27)	0.005	1.72 (1.06–2.78)	0.028
Model 4^d^	1.45 (0.86–2.46)	0.164	3.10 (1.44–6.68)	0.004	1.89 (1.15–3.11)	0.012

Logistic regression analysis was performed. BMI, body mass index; HDL-C, high-density lipoprotein cholesterol; OR, odds ratio; CI, confidence interval.

a Unadjusted.

b Adjusted for age and BMI.

c Adjusted for all covariates in model 2 plus triglyceride and HDL-C.

d Adjusted for all covariates in model 3 plus alcohol consumption and smoking status.

## Discussion

Atrophic gastritis increased the likelihood of osteoporosis in postmenopausal women over 60 years of age. The association remained significant even after controlling for anthropometric, laboratory, and social variables. To the best of our knowledge, this is the first report that evaluates the relationship between atrophic gastritis and osteoporosis in the elderly over 60 years of age.

Osteoporosis poses a great threat to the aging society. Aging is accompanied by the risk of osteoporosis and associated fractures, thereby causing increased disability-adjusted life years lost worldwide [2]. Aside from aging, osteoporosis and osteoporotic fractures have many other risk factors. Females are at high risk for osteoporosis. The peak bone mass of women was lower than that of men, and the BMD levels of postmenopausal women decrease abruptly after menopause because of a lack of estrogen [16], [17]. Cigarette smoking increases postmenopausal bone loss [5], and heavy alcohol drinking exerts a negative effect on bone health [6]. Bone serves as a reservoir for the storage of calcium, and vitamin D plays a critical role in gastrointestinal calcium absorption. Therefore, vitamin D deficiency [7] and low dietary calcium [8] facilitate bone loss and osteoporotic fractures.

Along with other risk factors for osteoporosis, hypochlorhydric or achlorhydric conditions, including gastrectomy, and the use of antacids are important because the dissolution and absorption of calcium salts decrease in non-acidic conditions [9], [11], [18]. Stomach resection adversely affected bone metabolism and decreased BMD even in a partial gastrectomy group [19]. Gastrectomy, including bariatric surgery, increases the risk of osteoporosis and fractures due to weight loss and change of body composition as well as calcium malabsorption [10], [18]. In rats subjected to gastrectomy and fundectomy, blood calcium concentration decreased slightly within three weeks after surgery, reflecting an impaired capacity of converting insoluble calcium into soluble calcium salts [20]. Acid-suppressive medication use could also raise the risk of fractures. A meta-analysis revealed that proton-pump inhibitors increase the risk of hip, spine, and any-site fractures by 30, 56, and 16%, respectively [11]. Long-term use of proton-pump inhibitors markedly increased the risk of hip fractures in another study [21].

Atrophic gastritis, which is more prevalent in the elderly and associated with *H. pylori* infection [22], is characterized by the loss of an appropriate number of glands in the gastric mucosa [23] and therefore causes a hypochlorhydric or achlorhydric stomach. As a result, the absorption of minerals and vitamins could be hampered; however, the relationship between the presence of atrophic gastritis and micronutrient absorption has been poorly studied [9]. Moreover, studies about atrophic gastritis and osteoporosis are rare and inconclusive [12], [13]. We found that atrophic gastritis is associated with osteoporosis in postmenopausal women aged 60 or older after adjusting for age, BMI, TG, HDL-C, alcohol consumption, and smoking status. Previous studies reported that there was no relationship between BMD and atrophic gastritis [13], [24]; however, the participants in these studies were relatively young women below the age of 60. Aging is related to the presence and the progression of atrophic gastritis and intestinal metaplasia [22], [25]. In a Korean study, the prevalence of atrophic gastritis was more than 50% in antrum and 23.5% in body for those older than 60 [22], which was in accordance with our result (56.9% and 43.1% respectively in osteoporotic and non-osteoporotic participants, Table 1). One of the possible reasons that no relationship between BMD and atrophic gastritis was seen in these studies is that the ability to produce acid in relatively young participants remained, and moderate concentrations of acid secretion would be enough to induce reasonable calcium absorption in the small intestine. In addition, the study sample of these studies had a heterogeneous nature, including different ethnicities [24], and the sample sizes in these studies were small. The present study had a larger sample size and only included women over 60 years of age of a single population (native Korean). In a study of pepsinogen I and BMD, decreased lumbar spine BMD correlated with a lower serum level of group 1 pepsinogens [12]. The concentration of pepsinogen I indicates the functional ability of the oxyntic mucosa, and thereby, a decreased pepsinogen I level is a marker of mucosal atrophy [26]. This result is in agreement with our results, supporting the hypothesis that the grade of atrophy in the oxyntic mucosa is linearly correlated with the BMD.

Our report had several strengths, including its relatively large sample size, and that it was drawn from a homogenous population of elderly women over 60 years of age. However, this study also had some weaknesses. First, the design of this study is cross-sectional, not allowing for detection of causality. Second, the diagnosis of atrophic gastritis was based on endoscopic findings, not on biopsy specimens due to the nature of routine health check-ups. In recent publications, however, a correlation was found between endoscopic and histological findings in diagnosing atrophic gastritis in Korean samples [27], [28]. The young age group (below 50 years) was associated with decreased sensitivity of endoscopic diagnosis of atrophic gastritis in a study [28], which infers that the sensitivity was fair in the elderly. We included only women over 60 years of age in the study to avoid uncertainty in the diagnosis of atrophic gastritis. Third, we only checked the status of *H. pylori* infection from some participants due to the nature of health examinations, and we did not assess the use of drugs such as proton-pump inhibitors or antibiotics that can affect biopsy results for *H. pylori*. Finally, we did not check the use of anti-acid medications such as proton-pump inhibitors or histamine 2 blockers that can affect bone mineral density.

In conclusion, atrophic gastritis is associated with an increased odds of osteoporosis in elderly women after adjusting for anthropometric, laboratory, and social parameters. Further studies are needed to identify our conclusions, which need to be confirmed in studies with a prospective design, larger sample, and other populations.

## Supporting Information

Table S1 **Data of atrophic gastritis, bone mineral density, and other related variables.**
(XLS)
